# Role of Citrullinated Collagen in Autoimmune Arthritis

**DOI:** 10.3390/ijms23179833

**Published:** 2022-08-30

**Authors:** Linda K. Myers, Ying-Xin Ouyang, Jay R. Patel, Herman H. Odens, Virginia Woo-Rasberry, Jeoungeun Park, Ae-Kyung Yi, Edward F. Rosloniec, David D. Brand, John M. Stuart, Andrew H. Kang

**Affiliations:** 1 Department of Pediatrics, University of Tennessee Health Science Center, 50 N. Dunlap, Rm. 461R, Memphis, TN 38103, USA; 2 Department of Medicine, University of Tennessee Health Science Center, 956 Court Ave., Memphis, TN 38163, USA; 3 Department of Microbiology-Immunology-Biochemistry, University of Tennessee Health Science Center, 858 Madison Ave., Memphis, TN 38163, USA; 4 Memphis Veterans Affairs Medical Center, 1030 Jefferson Ave., Memphis, TN 38104, USA; 5 Department of Pathology and Laboratory Medicine, University of Tennessee Health Science Center, 930 Madison Ave., Memphis TN 38163, USA

**Keywords:** post-translational modifications, inhibitory receptors, autoimmunity, collagen, inflammation

## Abstract

Citrullination of proteins plays an important role in protein function and it has recently become clear that citrullinated proteins play a role in immune responses. In this study we examined how citrullinated collagen, an extracellular matrix protein, affects T-cell function during the development of autoimmune arthritis. Using an HLA-DR1 transgenic mouse model of rheumatoid arthritis, mice were treated intraperitoneally with either native type I collagen (CI), citrullinated CI (cit-CI), or phosphate buffered saline (PBS) prior to induction of autoimmune arthritis. While the mice given native CI had significantly less severe arthritis than controls administered PBS, mice receiving cit-CI had no decrease in the severity of autoimmune arthritis. Using Jurkat cells expressing the inhibitory receptor leukocyte-associated immunoglobulin-like receptor-1 (LAIR-1), Western blot analysis indicated that while CI and cit-CI bound to LAIR-1 with similar affinity, only CI induced phosphorylation of the LAIR ITIM tyrosines; cit-CI was ineffective. These data suggest that cit-CI acts as an antagonist of LAIR-1 signaling, and that the severity of autoimmune arthritis can effectively be altered by targeting T cells with citrullinated collagen.

## 1. Introduction

Collagen, an extracellular matrix protein, is the most abundant type of protein in vertebrates and is essential for the health of the human body [1]. However, its role in regulating immune function is not as well known. When extracted, purified, and administered systemically, both human and murine studies have shown that it attenuates autoimmune arthritis [2,3]. This suppression is partly due to the induction of immune tolerance, which is the prevention of an immune response against a particular antigen, by anergy, deletion, or induced Tregs [4]. Another mechanism which has been less explored is the activation of the inhibitory receptor Leukocyte-Associated Immunoglobulin-Like Receptor-1 (LAIR-1) [5]. Since collagen is a natural ligand for LAIR-1, its engagement on immune cells downregulates excessive inflammation [1].

Emerging identification of the structural properties of collagen that influence its function illustrate the importance of posttranslational modifications (PTMs), such as citrullination in shaping disease states. Citrullination [6,7] is the conversion of arginine residues into citrulline residues [8], a process catalyzed by the enzyme peptidylarginine deiminase (PADI) [9]. Although citrullination is involved in protein degradation by interrupting molecular interactions mediated by positively charged arginine residues [8], citrullinated proteins have also been found in the synovium of rheumatoid arthritis (RA), becoming major targets of autoantibodies in patients with RA. Autoantibodies that react with citrullinated CII (cit-CII) have been identified in the sera and joint fluids of RA patients and they enhance autoimmunity by binding cartilage via recognition of immune epitopes on CII [10].

In this study, we gained new insights into the mechanisms by which citrullination of collagen controls autoimmune arthritis. We used the well-studied mouse model of arthritis collagen-induced arthritis (CIA) because it mimics RA [11]. After the PADi4 enzyme was employed to citrullinate collagen, the resulting proteins were tested for binding and activation of LAIR-1. The cit-collagen was then administered to mice to determine whether it altered the severity of autoimmune arthritis. We believe the new understandings gained by these studies will lead to more precise therapies for arthritis.

## 2. Results

### 2.1. Collagen-Induced Suppression of Arthritis Depends on LAIR-1

**Figure 1:**
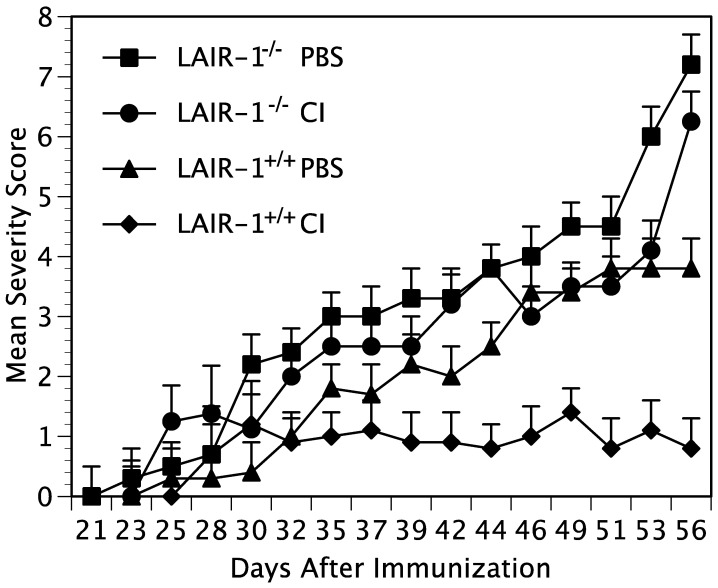
LAIR-1 mediates suppression of CIA. Two groups (*n* = 10 per group) of DR1/LAIR-1^+/+^ mice and two groups of littermate DR1/LAIR-1^−^^/−^ mice were immunized with type II collagen/CFA. Then, each mouse was administered intraperitoneally a dose of either 100µg CI or PBS weekly for four weeks beginning one week after immunization. Each mouse was scored three times weekly beginning 2 weeks post immunization. The severity of the arthritis was statistically significant comparing CI treatment with PBS in LAIR-1^+/+^ mice beginning on day 44 (*p* ≤ 0.05) post immunization and continuing until the end of the experiment on day 56. (*p* ≤ 0.001).

In order to determine how effectively collagen regulates immune function, we administered either CI or PBS intraperitoneally to mice that had previously been immunized with CII/CFA to induce arthritis. Two groups of DR1/LAIR-1^+/+^ mice and two groups of littermate DR1/LAIR-1^−/−^ mice were immunized with CII/CFA, followed by intraperitoneal administration of either 100 µg CI or PBS weekly for four weeks beginning one week after immunization. When mice were observed for the development of arthritis, the LAIR-1^+/+^ mice receiving the CI had significantly less severe arthritis compared to littermate controls administered PBS (Figure 1). This severity was statistically significant beginning on day 44 (*p* ≤ 0.05) after immunization and continuing until the end of the experiment on day 56 (*p* ≤ 0.001). On the other hand, the LAIR-1^−/−^ mice were unaffected by the collagen, despite undergoing the same treatments as the LAIR-1^+/+^ mice. Their arthritis was comparable to the controls treated with PBS (Figure 1). Taken together, these data demonstrate that LAIR-1 is critical for the suppression of arthritis induced by systemic administration of collagen.

### 2.2. Citrullination of Type I and Type II Collagen

**Figure 2:**
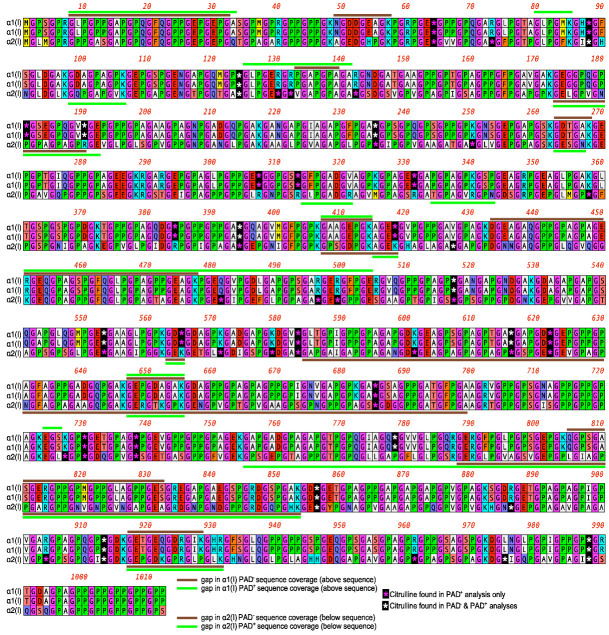
Citrullination of type I collagen. The citrullination patterns of α1(I), and α2(I) are shown. CI was treated with PADi4 and analyzed by mass spectrophotometry for the presence of citrullines. The white stars indicate citrullines that were present before and after treatment with PADi4. The red stars indicate citrullines only present after treatment with the enzyme.

**Figure 3:**
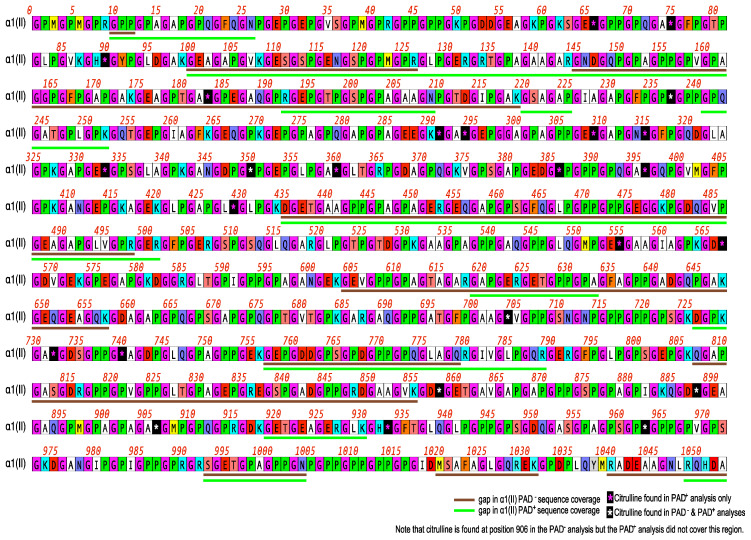
Citrullination of type II collagen. The citrullination pattern α1(II) is shown. CI and CII were treated with PADi4 and then analyzed by mass spectrophotometry for the presence of citrullines. The white stars indicate citrullines that were present before and after treatment with PADi4. The red stars indicate citrullines only present after treatment with the enzyme.

CI and CII were each cultured with PADi4, and the resulting collagens were analyzed by mass spectrophotometry. The results of the mass spectrophotometry analysis following the culture of PADi4 with CI are shown in Figure 2; [α1(I), α2(I)] and after culture with CII in Figure 3; [α1(II)]. These data show that α1(I) collagen had a threefold increase in citrullination (11 to 36 citrullines); α2(I) collagen had a four-fold increase (8 to 34 citrullines); and α1(II) collagen had a two-and-a-half-fold increase (9 to 23 citrullines). The resulting hyper-citrullinated collagens were selected for functional testing in vitro.

### 2.3. Cit-Collagen Cannot Activate LAIR-1

**Figure 4:**
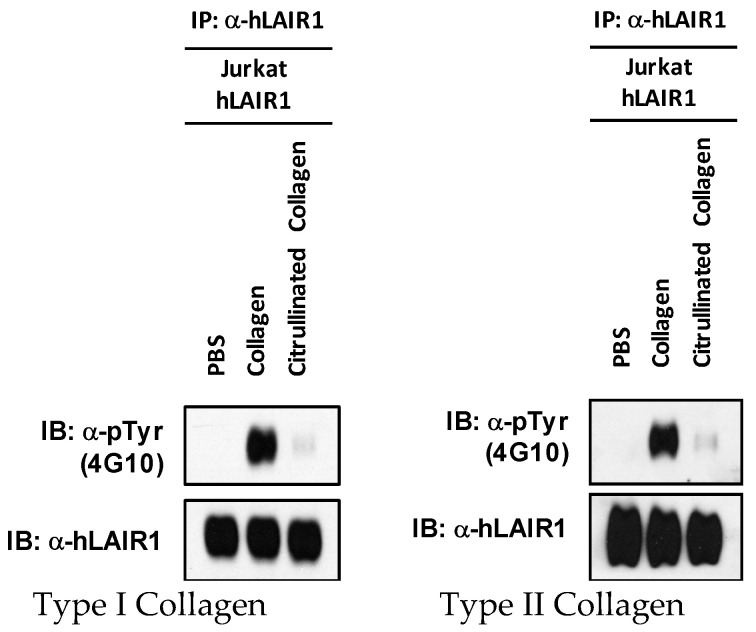
Phosphorylation of human LAIR1 by citrullinated collagen. Jurkat cells that overexpress the human LAIR1 (hLAIR1) were stimulated with PBS, collagen (200 µg/mL), or citrullinated collagen (200 µg/mL) for 5 min. Human LAIR1 was immunoprecipitated from 500 µg of total proteins by anti-human LAIR1 antibody (α-hLAIR1 (NKTA255), Thermo Fisher) using protein A/G bead. Phosphorylation of human LAIR1 was examined with the anti-phosphotyrosine antibody (α -pTyr 4G10, Millipore Human LAIR1 was used as control. The left panel represents type I collagen, and the right panel represents type II collagen. Data are representative of three separate analyses.

Collagen is a natural ligand for the inhibitory receptor, LAIR-1. Although LAIR-1 is expressed on many immune cells, we have chosen to focus initially on the CD4+ T cell because CD4+ T cells are dysregulated in RA. Cells from the human T-cell line Jurkat, which expresses LAIR-1, were treated with both citrullinated and uncitrullinated CI or CII. LAIR-1 proteins in whole-cell lysates were immunoprecipitated and phosphorylation was measured by Western blot analysis. As shown in Figure 4, when LAIR-1 was activated by the ligands, CI or CII, phosphorylation of the LAIR ITIM tyrosines readily occured within five minutes. However, citrullinated ligands, cit-CI or cit-CII, induced no detectable activation. Taken together, these data suggest that citrullination of collagen abrogates the ability of collagen to activate LAIR-1 in CD4+ T-cells.

### 2.4. Cit-Collagen Acts an Antagonist of LAIR-1

**Figure 5:**
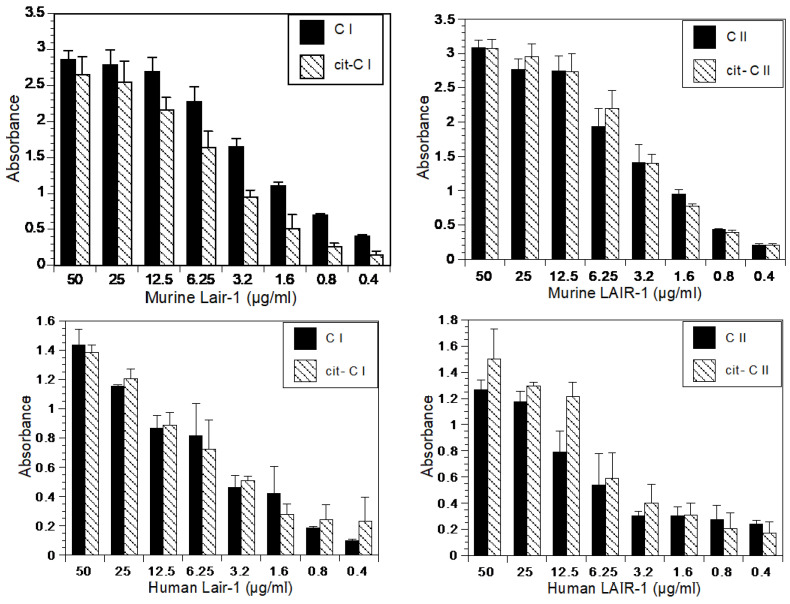
Cit-Collagen binds to LAIR with similar affinity as native Collagen. Wells were coated with CI, cit-CI, CII, or cit-CII before adding recombinant LAIR-1 (murine LAIR-1 in the (**upper panel**) and human LAIR-1 in the (**lower panel**)) at the indicated concentrations. Binding was detected using an HRP conjugated α -murine LAIR-1 ab, or an α-human LAIR-1 ab followed by a biotinylated α IgG and streptavidin -HRP with reading of absorbance at 450. The control protein, ovalbulmin did not bind the collagens murine LAIR-1 or human LAIR-1.

One possibility is that citrullination might prevent the physical interaction of collagen with the receptor LAIR-1. To test this possibility, LAIR-1 binding assays were performed. As shown in Figure 5, LAIR-1 affinity for cit-CI and cit-CII was equivalent to that of CI and CII for both human and murine LAIR-1. The control protein ovalbulmin did not bind the collagens murine LAIR-1 or human LAIR-1. These data suggest that cit-collagen acts as an antagonist of LAIR-1, binding to the receptor while preventing its activation. Therefore, cit-collagen at the site of inflammation effectively blocks LAIR-1 downregulation of the autoimmune response.

### 2.5. Treatment of Autoimmune Arthritis with Citrullinated Collagen

**Figure 6:**
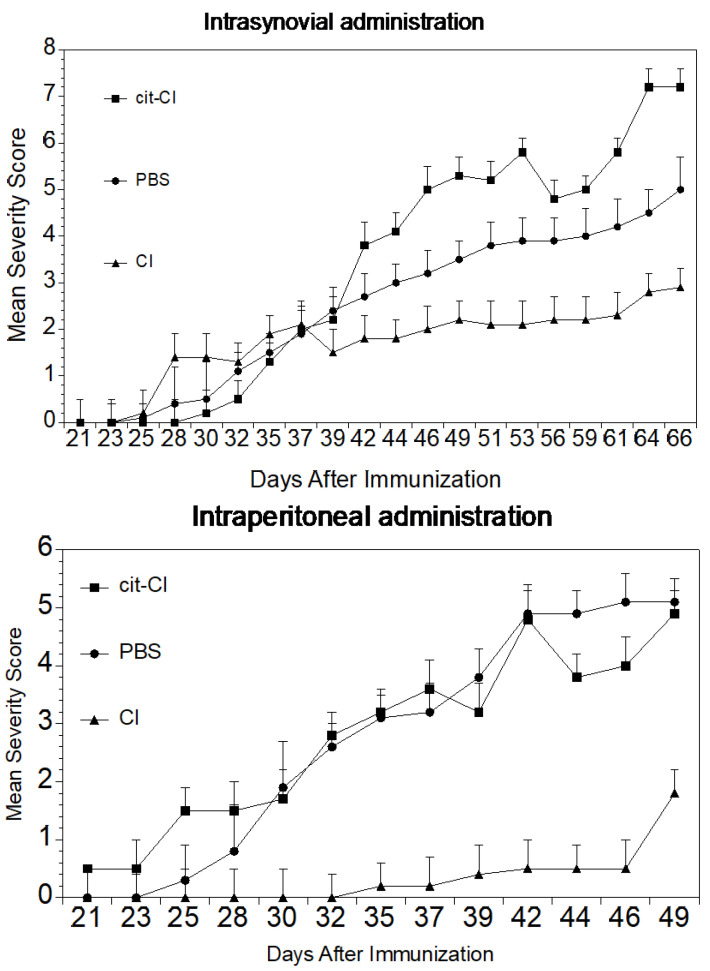
Treatment of arthritis with citrullinated collagen. (**Upper panel**). DR1 mice (*n* = 10 per group) were immunized with CII/CFA to induce arthritis and were injected intrasynovially in the hindpaws with either cit-CI or CI. Mice were scored three times weekly and evaluated for the severity of arthritis. The arthritis was more severe in mice given cit-CI intrasynovially compared to mice treated with CI. (**Lower panel**). DR1 mice were treated weekly intraperitoneally with either CI, cit-CI, or PBS. As above, CI could significantly reduce the severity of arthritis in the DR1 mice, while the mice treated with PBS or cit-CI developed arthritis at an expected rate.

Our priority was to establish how citrullination of collagen affects its proficiency in suppressing autoimmune arthritis when administered therapeutically. In order to better determine its efficacy, two different routes of delivery were tested; intrasynovial and intraperitoneal. DR1 mice were immunized with CII/CFA to induce arthritis prior to three intrasynovial injections in the hindpaws with either cit-CI or CI. When mice were evaluated for the severity of arthritis, the CI induced suppression whereas the cit-CI was ineffective (Figure 6). In a second therapeutic protocol, DR1 mice were immunized with CII/CFA to induce arthritis and were treated weekly for four weeks intraperitoneally with either CI, cit-CI, or PBS. Again, CI significantly reduced the severity of arthritis in the DR1 mice, while the mice treated with PBS or cit-CI remained severely arthritic. Although the group of mice were too small to determine whether one route of delivery was superior to the other, these data clearly show that collagen given either intrperitoneally or intrasynovially therapeutically can reduce autoimmune arthritis. On the other hand, collagen loses its potency if citrullinated prior to administration by either route.

## 3. Discussion

Collagen, an extracellular matrix protein, plays an important role in regulating immune function. In this study, the collagen-induced arthritis (CIA) murine model was used to examine the mechanism by which the severity of autoimmune arthritis is regulated by collagen and how citrullination affects this pathogenesis. CIA shares many features with RA, such as synovial hyperplasia, mononuclear cell infiltration, degradation of cartilage, and linkage to expression of specific MHC class II genes, making it a useful model for these studies. The MHC Class II gene HLA-DRβ1*0101 was selected because it enhanced the susceptibility to autoimmune conditions such as RA.

Citrullination is the posttranslational conversion of a gene-encoded arginine molecule into the non-encoded amino acid citrulline. This process (deimination) is mediated by peptidyl arginine deiminases (PADi) in a calcium-dependent manner. Under physiological conditions in cells, PADis are inactive until stimulated with calcium. Once stimulated, these enzymes citrullinate a number of structural proteins, including collagen. At the protein level, this modification is a hydrolytic reaction and causes an increase of 1 Da mass together with the loss of one positive charge, changing both the charge and hydrogen bonding capacity of the protein [12]. Although several PAD enzymes exist in humans, PADi4, located in inflammatory cells (macrophages, eosinophils, and neutrophils), mammary gland cells, and tumors, was the most effective in inducing citrullination of collagen in vitro.

The location of the arginine affects whether or not it is citrullinated. In general, arginines next to aspartic acid residues are citrullinated 80–90% of the time, while they are citrullinated only 0–5% of the time when next to glutamic acid and poorly citrullinated when next to an amino group or flanked by a proline residue [12,13]. Most of the citrullinated arginines in our samples were located near aspartic acid and glutamic acid residues, although some citrullinated arginines were flanked by prolines.

Under physiological conditions, PADis are inactive [12]. However, PADis become activated when calcium levels increase above the physiological concentration, such as during apoptosis or terminal epidermal differentiation. Once the PADis seep out of the cells, they target extracellular proteins in the joints such as fibrin and antithrombin. The resulting citrullination allows cell movement, so that inflammatory cells and platelets travel into the extracellular matrix to allow wound healing. Although PADis maintain many vital cellular processes, such as gene regulation (chromatin relaxation and activation of transcription), plasticity of CNS [13,14], immune responses [15], and cell apoptosis [16], this delicate balance can become dysregulated [12] when there is tissue injury or variation in thickness due to elasticity. Citrullinated proteins in excess can become autoantigens, activating immune responses, resulting in the production of anti-citrulline antibodies. The hypercitrullination observed in periodontitis mirrors the citrullinome of RA, which is most likely induced by *Aggregatibacter actinomycetemcomitans* through its pore-forming toxin, leukotoxin A [17], or by the prokaryote PAD-expressing oral pathogen *Porphyromonas gingivalis* [18].

Citrullination is felt to contribute to the development of several autoimmune diseases such as rheumatoid arthritis [19], cancer [20,21], and diabetes [22,23]. In RA, PADi2- and PADi4-secreting leukocytes infiltrate into the chronically inflamed synovia of patients [17]. As a result, RA has a distinct citrullinated antigen profile [8]. These autoantibodies are detectable in the early phases of RA in patients, with high specificity, thereby making these useful biomarkers for the disease. In animal models, PAD enzyme inhibition has been shown to ameliorate collagen-induced arthritis [24] and four single nucleotide polymorphisms (SNPs) have been identified in the exons of the PADI type 4 (PADI-4) gene associated with the severity of RA. In addition, the presence of the human RA susceptibility allele expressed as a transgene in our B6.DR1 mice rendered them capable of producing anti-citrullinated peptide antobodies during CIA development [18,25]. Similarly, patients with multiple sclerosis (MS), have increased levels of citrullinated myelin basic protein, which leads to demyelination of the myelin sheath reducing nerve signal transduction [26]. Histone citrullination by PAD4 in neutrophils is involved in neutrophil extracellular trap (NET) formation [27], a common feature of the early innate immune response to bacteria and fungi. The PAD hyperactivation causing NETs and adverse effects has been observed in several inflammatory lung diseases, including cystic fibrosis (CF) [28].

In this manuscript, we explore new mechanisms by which the immune response against a particular antigen, such as collagen, can be downregulated [4]. Tolerance of self-antigens prevents potentially harmful immune responses against the body’s own proteins, through anergy, deletion, or development of induced Tregs [4]. These regulatory immune cells circulate throughout the body to maintain tolerance, and are important for turning an immune response off after a problem is resolved. However, our data suggest there is another important mechanism by which the inflammation induced by citrullinated proteins can be attenuated. Collagen administered intraperitoneally can activate the inhibitory receptor LAIR-1 (CD305), a transmembrane glycoprotein receptor with an extracellular Ig-like domain and two immune-receptor tyrosine-based inhibition motif (ITIM) tails. It is expressed and can be activated on many immune cells. Therefore, we have chosen to focus initially on the CD4+ T cell, because LAIR-1 can be upregulated on CD4^+^ T cells, and CD4+ T cells, especially Th1 and Th17 cells, play a prominent role in the initiation of systemic immune responses and are dysregulated in RA. Traditionally, inhibitory receptors bind to ligands bound to other cells, but LAIR-1 is somewhat unique in that collagen, both transmembrane and/or extracellular matrix, is a high affinity ligand for LAIR-1 [29]. Collagen binding to the LAIR-1 receptor will result in activating downstream phosphatases (SHP-1 and SHP-2) and kinases (C-terminal Src kinase) that act as negative regulators for antigen-dependent activation/growth of T cells [29].

Our data clearly show that the effectiveness of LAIR-1 was hindered when the collagen was citrullinated. The citrullinated molecules bind to the active site and prevent binding of the actual ligand, collagen, resulting in an inactive LAIR-1. We believe that if citrullination could be inhibited or if citrullinated collagen could be eliminated, the immune system could more effectively shut down inflammation. An understanding of the mechanisms underlying tolerance and citrullination promises opportunities to reprogram the immune system so that more effective treatments for autoimmune arthritis can be developed.

## 4. Materials and Methods

### 4.1. Antibodies

In western blot studies, the anti-human LAIR1 and the anti-phospho-tyrosine specific antibodies were purchased from R&D Systems, Inc. (Minneapolis, MN, USA). For the binding studies, an HRP-conjugated monoclonal antibody against murine LAIR-1 (Novus #NBP1-43324H) Novus Biologicals, (Centennial, CO, USA) and a monoclonal antibody against human LAIR-1 (R&D #2664) (R&D Systems, Inc. Minneapolis, MN, USA) were used. An anti-citrulline antibody (#AB5612) came from EMD Millipore, (Danvers, MA, USA). The secondary antibodies used were goat anti-rabbit SKU: 08674371 and goat anti-mouse SKU: 0855550 from MP Biomedicals (Santa Ana, CA, USA).

### 4.2. Other Reagents

PADI (types 2 and 4) were purchased from Cayman Chemical, (Ann Arbor, MI, USA), #10785 and #10500, respectively. PADI (types 1, 2, 3, and one isolated from rabbit skeletal muscle) were purchased from Sigma Aldrich, (St. Louis, MO., USA), #SRP0326, SRP0327, SRP0328, and P1584, respectively. Other reagents included Protein A/G plus agarose (Santa Cruz Biotech., Dallas, TX, USA, #sc-2003); SimplyBlue SafeStain #LC6060, (Pierce-Thermo Fisher, Waltham, MA, USA); and SuperSignal West Pico Plus Chemiluminescent Substrate #34577 Pierce Thermo-Fischer, (Waltham, MA, USA). All other chemicals were purchased from Sigma Aldrich, (St. Louis, MO, USA). Difco skim milk #232100 was purchased from BD Biosciences, (San Jose, CA, USA).

### 4.3. Preparation of Cartilage Derived Type II Collagen (CII) and Skin-Derived Type I Collagen

Native type I collagen was solubilized from bovine skin and native type II collagen was solubilized from fetal calf articular cartilage by limited-pepsin digestion and purified as described earlier [11]. The purified collagen was dissolved in cold 10 mM acetic acid at 3 or 4 mg/mL and stored frozen at −70 °C until used. In some experiments, type I collagen from Advanced Biomatrix #5005 (Carlsbad, CA, USA) was used. α1(I), α2(I), and α1(II), represent the constituent protein chains of bovine CI and CII, respectively, isolated by carboxymethyl-cellulose chromatography.

### 4.4. Citrullination of Collagen

CI was cultured with the following panel of PADi enzymes: 1, 2, 3, 4, and a PADi isolated from rabbit skeletal muscle, to determine which was most effective. Our studies showed that PADi 4 was the most efficient in vitro, so it was used for all subsequent experiments shown in this manuscript. Briefly, 10 mg of collagen was dialyzed against 10 mM acetic acid with 3 changes, separately. Following dialysis, the collagens were denatured at 50 °C for 20 min, then placed into reaction buffer of 100 mM Tris/150 mM NaCl, pH 7.4. Calcium chloride and DTT were added to a final concentration of 10 mM and 2.5 mM, respectively. The collagen was separated into aliquots (control and for each PADi treated), and then the PADis were added at a concentration of 1 unit/mL for 0, 2, and 4 h at 37 °C and 50 °C. The reactions were stopped by the addition of EDTA. The PADis and auto-citrullinated PADis were excluded by spin column and immunoprecipitation using the citrulline antibody bound to Protein A/G agarose. The final collagen buffer was changed to PBS pH 7.4. Control collagen was treated identically except for the absence of PADi4.

### 4.5. SDS-PAGE and Western Blotting

Confirmation of citrullination by gel electrophoresis of 6.5% SDS-PAGE stained with Coomassie blue and immunoblotting probed with citrulline antibody were performed. Briefly, immunoblot was performed and blocked with 5% dry milk/TBS-T pH 7.4, then probed with citrulline antibody at 1:500 in 1% milk in TBS-T (Tris buffered saline-Tween, pH 7.4) overnight at 4 °C, and the secondary antibody at 1:5000 in TBS-T for 2.5 h at RT, and developed using SuperSignal West Pico Plus Chemiluminescent Substrate (ThermoFisher Scientific, Waltham, MA, USA).

### 4.6. Colorimetric Analysis of Citrullination

The amount of citrullination was also determined by colorimetric analysis as previously described [30]. Citrulline was used as the standard (Sigma Aldrich, St. Louis, MO, USA, #C7629). The analysis was performed on Nunc plates and read on Spectromax 384 (Molecular Devices LLC., San Jose, CA, USA) at 530 λ.

### 4.7. Detection of Citrullination by Mass Spectometry

Citrullinated and non-citrullinated samples were also analyzed by LC-MS/MS performed by MS Bioworks Protein Mass Spectrometry Services, Ann Arbor, MI, USA. Briefly, 1 µg of each sample was reduced with 10 mM dithiothreitol at 60 °C followed by alkylation with 15 mM iodoacetamide at RT. Proteins were digested with Lys-C (Promega) overnight at 37 °C and quenched with formic acid and desalted using an Empore SD solid-phase extraction plate. Samples were lyophilized and reconstituted in 0.1% TFA for analysis. The peptides were analyzed by nano LC-MS/MS with a Waters NanoAcquity HPLC system interfaced to a ThermoFisher Q Exactive mass spectrometer. They were loaded on a trapping column and eluted over a 75 µm analytical column at 350 nL/min; both columns were packed with Luna C18 resin (Phenomenex). A one-hour gradient was employed. The mass spectrometer was operated in data-dependent mode, with the Orbitrap operating at 60,000 FWHM and 17,500 FWHM for MS and MS/MS, respectively. The fifteen most abundant ions were selected for MS/MS.

The data processing used a Byonic with the parameters: Enzyme: None; Database: UniProt Bovine (concatenated forward and reverse); Fixed modification: Carbamidomethyl (C); Variable modifications: Oxidation (M), Acetyl (N-term), Pyro-Glu (N-term Q), Deamidation (N/Q/R); Mass values: Monoisotopic; Peptide Mass Tolerance: 10 ppm; and Fragment Mass Tolerance: 0.02 Da. The Mascot Dat files were parsed into Scaffold (Proteome Software) for validation, filtering, and to create a nonredundant list per sample. Data were filtered using a 1% protein and peptide FDR, requiring at least two unique peptides per protein. Scaffold results were exported as mzldentML and imported into Scaffold PTM in order to assign site localization probabilities using A-Score [31].

### 4.8. Animals

Mice expressing the chimeric (human/mouse) DRB1*0101 construct were produced as previously described [32]. They were extensively backcrossed to C57 BL/6 mice obtained from Jackson Laboratories (Bar Harbor, ME, USA), and bred in our animal core facility at the VA Medical Center, Memphis. We chose the B6.DR1 tg mice because of their increased susceptibility to develop CIA [32].

LAIR-1 KO (knockout) mice [33] were backcrossed to the B6.DR1 transgenic mice with a B6 background for 12 generations. Genomic DNA was obtained from blood samples and PCR was used to identify mice homozygous for either LAIR-1^−/−^ or LAIR^+/+^ and expressing the DR1 transgene. The LAIR-1^−/−^/B6.DR1 mice were found to have healthy phenotypes. All mice were fed standard rodent chow (Ralston Purina Co., St Louis, MO, USA) and water ad libitum. Sentinel mice were routinely tested for murine pathogens. The animal protocols were reviewed and approved by the Animal Care and Use Committees of the University of Tennessee Health Science Center (UTHSC) (16-106.0-B, approved 12 October 2017) and the Memphis VA Medical Center (325541-19, approved 19 October 2018). The mice used were 8 to 12 weeks old.

### 4.9. Immunizations and Treatment of Arthritis

The mice were immunized with bovine CII to induce arthritis. CII was dissolved in 10 mM cold acetic acid and emulsified with complete Freund’s Adjuvant (CFA). The mice were immunized subcutaneously at the base of the tail with 100 μg of CII.

In some experiments, the mice were administered intraperitoneally a dose of either 100 µg of native CI, α1(I), or cit-α1(I), or 100 µL of PBS weekly for four weeks beginning one week after immunization. In other experiments, the mice were injected intrasynovially into each hindpaw, either 10 µg of native CI, 10 µg α1(I), 10 µg cit-α1(I), or 10 µL of PBS on days 6, 13, and 20 after immunization.

### 4.10. Measurement of the Severity of Arthritis

The severity of arthritis was determined by visually examining each forepaw and hindpaw and scoring them on a scale of 0 to 4 as described previously [34]. Arthritis severity was assessed in each paw every other day by two observers (one of whom was blinded to treatment) using the following scale: 0 = no swelling or redness, 1 = slight swelling and redness, 2 = moderate swelling or redness, 3 = marked swelling and redness, and 4 = marked swelling and redness with some deformity. Photographs of typical paws for the scoring system we used are available online at the Hooke Laboratories website (https://hookelabs.com/services/cro/cia/MouseCIAscoring.html accessed on 1 June 2019). Each mouse was scored thrice weekly beginning 3 weeks post immunization and continuing for 8 weeks. The mean severity score (sum of the severity scores for the group on each day/total number of animals in the group) and percent of arthritic limbs (sum of the number of arthritic limbs/total number of limbs per group X 100) was recorded at each time point.

### 4.11. Generation of LAIR-1 Overexpressing Jurkat Cells

Retroviral vectors expressing wild type (WT) or various mutant forms of human LAIR-1, were developed as previously described [35]. Briefly, the coding region of each was amplified by PCR using human cDNA as a template and then inserted into a MSCV-IRES-GFP retroviral vector. A total of 1 × 10^7^/10 mL of HEK293T cells in D10 (DMEM + L-glu + Na-pyruvate + 10% FBS) medium were seeded in the morning. After 9 h, the cells were transfected with the MSCV construct encoding empty control, WT, or various mutant forms of human LAIR-1 and helper DNAs using the calcium method (10 µg MSCV + 3 µg 3240 helper DNA + 3 µg pSR αG (VSV-G envelop) + 450 µL endo-free H_2_O + 50 µL 2.5 M CaCl_2_ (in 10 mM HEPES buffer) + 500 µL 2× HEPES buffer). The next morning, the medium was changed with fresh D10 medium. Virus particles in D10 medium were harvested after 1 day and 2 days. The virus particles were 20× concentrated by ultracentrifugation (25,000 rpm, 90 min, 4 °C). A total of 5 × 10^4^/mL of Jurkat cells were infected with the MSCV-IRES-GFP retrovirus that expressed empty control, WT, or mutant form of human LAIR-1 in the presence of 8 mg/mL of polybrene. After 1 day, the medium was changed with fresh D10 medium. Further selection was done by GFP positive sorting. Six days later, the levels of LAIR-1 in these cells were analyzed by SDS-PAGE followed by Western blot analysis using anti-human LAIR1 antibodies (1:1000 dilution factor).

### 4.12. Analysis of Protein Phosphorylation

Lysates of whole Jurkat cells expressing LAIR-1 following stimulation with either CI, CII, cit-CI, or cit-CII were separated using SDS-PAGE gels and electrotransferred onto nitrocellulose membranes. After transfer, the membrane was blocked in tris-buffered saline with Tween 20 buffer (TBS-T) containing 5% bovine serum albumin (BSA) for 1 h and incubated for 2 h with 12hosphor-specific antibodies. The membrane was then incubated with a secondary antibody (Bio-Rad) for 1 h and subjected to Enhanced Chemiluminescence (ECL) detection (ECL2 Western Blot kit, #P180196X3, #P180196X3, Thermo Scientific/Pierce, Waltham, MA, USA) according to the manufacturer’s protocol. To detect protein levels, the membranes were stripped and reblotted with antibodies pan-specific for the proteins of interest.

### 4.13. LAIR-1 Binding Assays

LAIR-1 binding assays were performed by incubating various concentrations of recombinant murine LAIR-1 (R&D #4016-LR-050) or human LAIR-1 (R&D #2664-LR-050) from R&D Systems, Inc. (Minneapolis, MN, USA) overnight at 4 °C, on a 96-well microtiter plate initially coated with collagen (CI, cit-CI, CII, or cit-CII) 5 µg/mL and blocked with 5% BSA. Excess protein was removed by washing with PBS containing 0.05% Tween 20. The plates were treated with either an HRP-conjugated monoclonal antibody against murine LAIR-1 (Novus #NBP1-43324H) Novus Biologicals, (Centennial CO, USA) or a monoclonal antibody against human LAIR-1 (R&D #2664). In the experiments using human LAIR-1, the antibody treatment was followed by incubation with a biotinylated anti-mouse IGg and streptavidin HRP. After washing, the plates were developed with tetramethyl benzidine (TMB, Thermo Fischer Scientific, Waltham, MA, USA). Optical density was quantified with a microplate reader at 450 nm (µQuant, Scimetrics, Katy, TX, USA). All values are expressed as the means ± standard deviations of four separate analyses. The control protein, ovalbulmin, did not bind the collagens, murine LAIR-1 or human LAIR-1.

### 4.14. Statistics

The calculations to determine the statistical significance of the tests were carried out using the programs SAS and GraphPad Prism 4. Depending on the data, a one-way ANOVA or Student’s *t*-test analysis was performed. The comparison of the mean variable values with a distribution significantly different from normal in two unrelated groups was performed using the Mann–Whitney test, while in more than two unrelated groups, the Kruskal–Wallis test was conducted. A value of *p* ≤ 0.05 was considered statistically significant.

## 5. Conclusions

In this study, collagen type I was compared side-by-side with citrullinated type I collagen in an autoimmune animal model to determine how citrullination affects the efficacy of collagen in inducing immune tolerance and suppression of disease. Both intraperitoneal and intrasynovial injection of type I collagen suppressed arthritis while cit-CI was ineffective. This post-translational modification had a major effect on the effectiveness of the collagen. Moreover, our data suggest that citrullination of collagen interferes with the activity of the immunosuppressive receptor LAIR-1, which is activated by collagen, its natural ligand. Although citrullinated collagen could bind to LAIR-1, it could not activate it, therefore functioning as a competitive inhibitor interfering with the natural ligand’s ability to activate its own receptor. Since citrullinated collagen is commonly found at sites of inflammation, this finding is important for designing therapeutics. A better understanding of the mechanisms underlying the ways in which citrullination affects tolerance promises opportunities to use citrullinated proteins as therapeutic targets.

## Data Availability

The datasets used and/or analyzed during the current study are available from the corresponding author on reasonable request.
